# Outcomes of COVID-19 patients with acute kidney injury and longitudinal analysis of laboratory markers during the hospital stay: A multi-center retrospective cohort experience from Pakistan

**DOI:** 10.1097/MD.0000000000032919

**Published:** 2023-02-10

**Authors:** Muhammad Nadeem Ahsan, Muhammad Sohaib Asghar, Sadia Iqbal, Haris Alvi, Mohammed Akram, Basmah Fayyaz, Syeda Ghazala Irshad, Irfan Ullah, Sarosh Alvi, Zohaib Yousaf

**Affiliations:** a Department of Nephrology, Dow University of Health Sciences-Ojha Campus, Karachi, Pakistan; b Department of Internal Medicine, Dow University of Health Sciences-Ojha Campus, Karachi, Pakistan; c Department of Internal Medicine, Liaquat National Hospital and Medical College, Karachi, Pakistan; d Department of Internal Medicine, Kabir Medical College Gandhara University, Peshawar, Pakistan; e Teaching Faculty, University of Khartoum, Khartoum, Sudan; f Department of Internal Medicine, Reading Hospital – Tower Health, Reading, PA.

**Keywords:** AKI, CKD, COVID-19, ICU, mortality, outcomes

## Abstract

The frequency of acute kidney injury (AKI) in COVID-19 patients can be varied and related to worse outcomes in the disease population. AKI is common among hospitalized patients with COVID-19, particularly the ones needing critical care. This study was conducted in order to determine the outcomes of hospitalized patients with prolonged hospital stays who suffered from COVID-19 associated AKI. It was conducted as a multi-centered, retrospective, cohort study, and including all patients who were diagnosed on COVID-19 PCR. End-stage renal disease patients on hemodialysis were excluded. The cohort included 1069 patients, with 68% males, mean age of 56.21 years, and majority within 50 to 75 years age group (60%). Mean disease onset was 14.43 ± 7.44 days and hospital stay was 7.01 ± 5.78 days. About 62% of patients stayed in intensive care and 18% of them were on invasive ventilation. The mortality rate was 27%. Frequency of AKI was 42%, around 14% of them were resolving during hospital stay and other 28% worsened. The mortality rate was significantly higher with AKI (OR: 4.7, *P* < .001). Alongside AKI, concomitant liver dysfunction was also significantly contributing to mortality (OR: 2.5), apart from ICU stay (OR: 2.9), invasive ventilation (OR: 9.2), and renal replacement therapy (OR: 2.4). Certain laboratory markers were associated with AKI throughout in-hospital stay.

## 1. Introduction

Severe Acute Respiratory Syndrome-Coronavirus-2 is an irresistible viral disease caused by an intense respiratory disorder, bringing about more than 3.8 million deaths, and arising as a worldwide emergency. The cases and deaths of COVID-19 cumulated remarkably, with 271 million and 5.32 million revealed as of December 17, 2021.^[1]^ As of March 2022, the number of COVID-19 cases is about 80 million in the United States according to the CDC.^[2]^ The coronaviruses are single-stranded RNA viruses affecting not only humans but animals too. It was first portrayed by Bynoe and Tyrell in 1966, who found the infection among patients with influenza.^[3]^ COVID-19 is infamous to cause extrapulmonary manifestations alongside respiratory dysfunction causing its effect on, renal, neurologic, gastrointestinal, hematological, and mental locale, making it a multisystem illness.^[4]^ The second most organs impacted after the lungs is the kidney, expanding the risk of death in COVID-19 patients.^[5]^

The frequency of acute kidney injury (AKI) in COVID-19 patients goes from 0.5% to 35% and has been related to worse prognosis.^[6]^ TMPRSS2 (trans-membrane serine protease) and ACE2 (Angiotensin-converting enzyme 2) are fundamentally liable for the entry of SARS-COV-2 into various kidney cell types affecting renal function.^[7]^ AKI is common among hospitalized patients with COVID-19, particularly the ones under critical care. It relates to intense kidney injury and is associated with a high death ratio.^[8]^ Various variables meaningfully affect AKI including, inflammatory reaction related to immunologic injury, cardio-renal disorder, cell invasion, and hypovolemia, with numerous others are being investigated.^[9]^ Histopathology reports of AKI patients with COVID-19 displayed to have tubular damage, glomerulopathy, and necrosis.^[10]^ Accordingly, apprehension of the hidden process of kidney injury throughout COVID-19 is critical for early Acknowledgments of the harm and the execution of ideal treatment.

This study was conducted to determine the outcomes of hospitalized patients with prolonged hospital stays who suffered from COVID-19-associated AKI. Further, any patient with known chronic kidney disease (CKD) was assessed for acute on chronic renal injury and longitudinal analysis of laboratory markers throughout the hospital stay was reported.

## 2. Methods

This study was conducted as a multi-centered, retrospective, cohort study, including all patients who were diagnosed as COVID-19 positive via either nasopharyngeal or oropharyngeal swab for Polymerase chain reaction (PCR) in three leading institutions in the metropolitan city of Karachi, Pakistan. The three institutions namely Dow University Hospital, Liaquat National Hospital, and Ziauddin University Hospital provided the ethical approval and utilized a diagnostic kit that exploits the principle of real-time fluorescence (RT-PCR), USA-WA1/2020 (of stock concentration 2.8E + 05 TCID 50/mL), with a lower detection limit of 0.003 TCID 50/mL. The duration of the study included patients admitted from March 2020 up to March 2021. Nasal swabs were used in majority of the diagnosed study population. Once admitted, the patients were monitored for their disease course and baseline laboratory investigations, daily follow-up, and the outcome at discharge or death was reported. The basic clinical data (including demographics, comorbidities, and symptoms) were obtained through the chart review of medical records, and further laboratory markers were assessed through electronic hospital management information systems. The admitting and discharging renal profiles were documented in all the patients to identify the derangements during the hospital stay with or without previously known chronic kidney disease. However, end-stage renal disease patients who were on renal replacement therapy were excluded from the final analysis. Rest of the known chronic kidney disease patients (of any stage) who were not on renal replacement therapy were included.

The laboratory data included a complete blood picture profile including neutrophil to lymphocyte ratio (NLR) and other differential leucocyte counts, biochemistry panel including renal markers, liver functional enzymes, coagulation profile, electrolytes including calcium, magnesium phosphate, and other inflammatory markers of COVID-19. The statistical analysis was conducted by using the IBM SPSS Statistics version 25.0 (IBM Corp., Armonk, NY). All continuous variables were described as mean and standard deviation. Significance calculated by non-parametric distribution Mann–Whitney *U* test as guided by Shapiro–Wilk test for equal distribution of data. The descriptive statistics for categorical variables were counts and percentages. The comparison of categorical data was done either using the Chi-square test or Fisher’s exact test (if the expected count was less than 5 in more than 20% cells). The descriptive statistics for categorical variables were counts and percentages. A *P* value of <.05 was considered statistically significant (two-tailed). A multinomial regression model was adopted to determine the significant association of renal injury and mortality with all baseline and clinical data. Odds ratios (ORs) along with the 95% confidence interval were obtained with an appropriate *P* value to predict the disease outcomes and association with renal derangements. Kaplan–Meier survival curves with Log-rank test were reported.

With concomitant liver dysfunction, individuals with a previous history of chronic liver disease, or known history of hepatitis B or/C were not considered. Peritoneal dialysis patients were also excluded. AKI was determined based on extended KDIGO classification of renal injury in COVID-19 patients exclusively, as previously published.^[11]^ Staging of the kidney injury was also established with KDIGO/AKIN criteria. Further, acute on chronic kidney injury was also determined based on worsening of renal markers during the COVID-19 disease course. STROBES guidelines were conformed for reporting the findings.^[12]^

## 3. Results

Our study cohort included 1069 patients, with 68% males and a mean age of 56.21 years. Majority of study participants were 51 to 75 years of age (60%). Travel history or other known exposures were not frequent (as shown in Table 1). Being a healthcare worker (6.4%) was considered one of the known exposures. Clinical data suggested that the mean onset of disease was 14.43 ± 7.44 days while mean days spent in the hospital were 7.01 ± 5.78. Around 62% of the cohort were admitted to intensive care or transferred to/stayed in a high dependency unit at any point of their hospital stay. Out of those, 18% were having invasive modes of ventilation.

**Table 1:** Demographic data, clinical features, and risk factors of the study population (n = 1069).

Characteristics	Variables	Mean/percentage
Age	In years	56.21 ± 14.86
Age groups	<25 yr	2.9
	26–50 yr	31.4
	51–75 yr	59.5
	>75 yr	6.3
Gender	Male	67.8
	Female	32.2
BMI	kg/m^2^	26.53 ± 5.36
BMI categories	<18.5 kg/m^2^	7.4
	18.5–24.9 kg/m^2^	22.5
	25.0–29.9 kg/m^2^	44.3
	≥30.0 kg/m^2^	25.8
Travel history	Present	3.1
	Absent	96.9
Known exposure	Family member	2.9
	Work place	2.8
	Healthcare	1.9
	Travelling	0.5
	Unknown	91.9
Profession	Healthcare worker	6.4
	Non-medical	93.6
Duration of disease (since onset)	In days	14.43 ± 7.44
Length of hospital stay	In days	7.01 ± 5.78
Hospital stay	ICU/HDU	61.9
	Non-ICU/HDU	38.1
Mode of ventilation	Invasive	18.3
	Noninvasive	81.7
Comorbidities	Diabetes	40.8
	Hypertension	47.5
	COPD	1.6
	IHD	10.7
	CKD	9.4
	CLD	2.4
	Asthma	4.3
	Stroke	1.8
	Hypothyroidism	3.6
Clinical features	Dry cough	59.4
	Productive cough	40.6
	Fever	76.9
	Sore throat	10.0
	Chest pain	8.0
	Dyspnea	55.1
	Fatigue	20.0
	Nasal congestion	1.8
	Headache	2.4
	Myalgia/arthralgia	7.5
	Nausea	2.2
	Vomiting	2.8
	Diarrhea	4.6
	Abdominal pain	2.6
	Lethargy	9.3
Clinical outcomes	Mortality	27.1
	Survived/discharged	72.9
Renal outcomes	AKI	41.9
	No AKI	58.1
Nature of renal injury	Resolving AKI	13.9
	Worsening AKI	28.0
Stages of AKI (KDIGO)	Stage 1	22.1
	Stage 2	8.7
	Stage 3	11.1
Concomitant acute liver dysfunction	Present	8.0
	Absent	92.0
Renal replacement therapy	Hemodialysis	20.0
	No hemodialysis	80.0

AKI = acute kidney injury, BMI = body mass index, CKD = chronic kidney disease, CLD = chronic liver disease, COPD = chronic obstructive pulmonary disease, HDU = high dependency unit, ICU = intensive care unit, IHD = ischemic heart disease.

Among the comorbidities, hypertension (48%) and diabetes (41%) were the most frequently reported, followed by ischemic heart disease (11%). Notably, 9% of the patients had known chronic kidney disease (any stage but not on renal replacement therapy). With symptomatology, fever (77%), dyspnea (55%), and fatigue (20%) other than cough were the most frequent. Dry cough (59%) was more likely to present than productive cough (41%), although some patients (13%) exhibited both. Coming to the outcome data, 27% of the patients did not survive and the rate of AKI in the whole cohort was 42%. Among those, 14% manifested resolving AKI during the hospital stay while the rest 28% worsened. Concomitant liver dysfunction was identified in further 8% of the cohort who had developed AKI. Hemodialysis was required as a modality of renal replacement therapy in 20% of the cohort.

**Figure 1.**
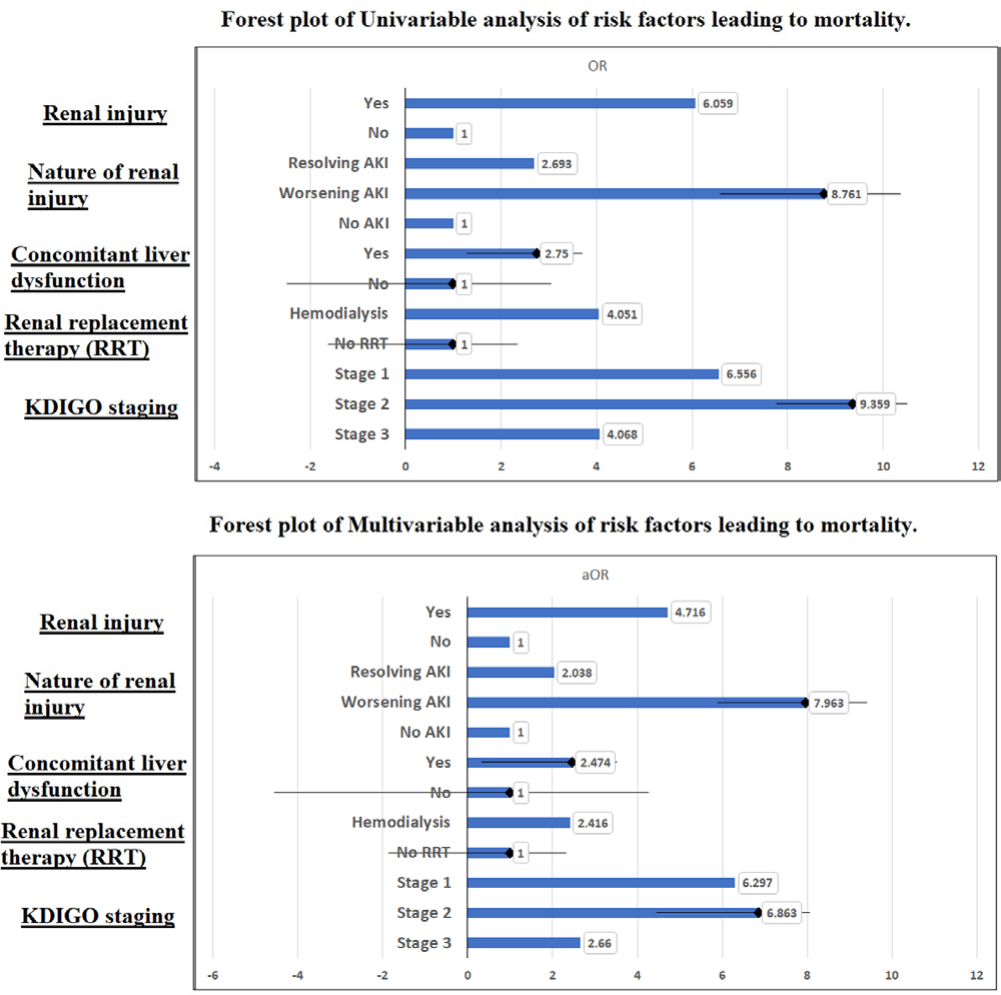
Forest plots representing univariate and multivariate analysis of risk factors associated with mortality. AKI = acute kidney injury, KDIGO = Kidney Disease: Improving Global Outcomes.

Factors that were associated with acute renal injury were showcased in Table 2. Young or middle age, presence of travel history, and sore throat were associated with a lower likelihood of AKI, while intensive care unit (ICU) stays (OR: 2.8), invasive ventilation (OR: 1.8), and hypertension (OR: 2.0) were significant predictors of AKI on multivariate analysis. However, the strongest association was with already known chronic renal disease (OR: 10.8). Therefore, acute on chronic kidney disease was a potential confounder in this cohort. But it was not associated with mortality as shown in Table 3. Factors associated with mortality in this cohort included invasive ventilation (OR: 9.2), ICU stay (OR: 3.0), productive cough (OR: 3.2), chest pain (OR: 3.5), and dyspnea (OR: 4.6). AKI leading to mortality was another significant factor with an adjusted odds of 4.7 (3.3–6.8) and worsening AKI during hospital stay was highly significant (OR: 7.963 [4.690–13.522]) as shown in Figure 1. Alongside AKI, concomitant liver dysfunction was significantly contributing to mortality (OR: 2.47) highlighting multi-organ involvement as a poor prognostic factor. Despite hemodialysis representing a high-risk group with declining renal function, the mortality rate is expectedly high (OR: 2.416 [1.208–4.835)], but it is clear that odds ratio reduced from 4.0 (AKI) to 2.4 (mortality), which can be indicative that hemodialysis in AKI reduced some mortality. But hemodialysis is a confounder when KDIGO staging was used to determine mortality. As we can see, in stage 1 AKI 37% patient ultimately needed hemodialysis, which increases to 49% in stage 2 and 86% in stage 3. Now, if we see the odds of mortality after adjusting for hemodialysis, stage 1 (aOR: 6.3) and stage 2 (aOR: 6.8) are associated more with mortality than stage 3 (aOR: 2.6) which is indicative of hemodialysis been more effective in these high risk patients and reducing mortality, despite being on dialysis have higher odds of mortality when compared to non-AKI group (OR: 2.4). Mortality was also highest in stage 2 AKI (57%), followed by 37% and 48% in stage 1 and stage 3 patients.

**Table 2:** Multivariable analysis of risk factors leading to renal injury (n = 1069).

Factors	Univariate		Multivariate	
	Crudes OR (95% CI)	*P* value	Adjusted OR (95% CI)	*P* value
Age (in yr)				
<25	0.217 (0.073–0.646)	**.006**	0.284 (0.092–0.879)	**.029**
26–50	0.369 (0.214–0.635)	**<.001**	0.458 (0.258–0.815)	**.008**
51–75	0.900 (0.539–1.502)	.686	0.942 (0.550–1.613)	.828
>75	1.000	–	1.000	–
Gender				
Male	1.129 (0.864–1.475)	.374	1.172 (0.872–1.576)	.292
Female	1.000	–	1.000	–
BMI (kg/m^2^)				
<18.5	1.018 (0.602–1.722)	.947	1.127 (0.658–1.931)	.664
18.5–24.9	1.052 (0.643–1.720)	.840	1.201 (0.726–1.988)	.476
25.0–29.9	1.171 (0.699–1.962)	.549	1.281 (0.755–2.175)	.359
≥30.0	1.000	–	1.000	–
Travel history				
Present	0.110 (0.014–0.843)	**.034**	0.075 (0.010–0.589)	**.014**
Absent	1.000	–	1.000	–
Profession				
Healthcare worker	0.690 (0.217–2.188)	.528	0.505 (0.120–2.122)	.351
Non-medical	1.000	–	1.000	–
Hospital stay				
ICU/HDU	3.627 (2.730–4.819)	**<.001**	2.785 (2.052–3.780)	**<.001**
Non-ICU/HDU	1.000	–	1.000	–
Mode of ventilation				
Invasive	2.251 (1.633–3.103)	**<.001**	1.660 (1.168–2.360)	**.005**
Noninvasive	1.000	–		
Comorbidities				
Diabetes	1.778 (1.059–2.983)	**.029**	1.552 (0.901–2.673)	.113
Hypertension	2.207 (1.313–3.710)	**.003**	1.999 (1.149–3.478)	**.014**
COPD	0.507 (0.052–4.945)	.559	0.385 (0.030–4.993)	.465
IHD	1.492 (0.669–3.330)	.328	1.065 (0.458–2.480)	.883
CKD	12.937 (3.743–44.718)	**<.001**	10.801 (2.856–40.850)	**<.001**
CLD	1.025 (0.168–6.246)	.979	1.824 (0.240–13.871)	.561
Asthma	1.281 (0.380–4.319)	.689	1.087 (0.245–4.828)	.913
Clinical features				
Dry cough	1.023 (0.705–1.484)	.904	1.188 (0.628–2.245)	.596
Productive cough	1.432 (0.761–2.693)	.265	0.818 (0.326–2.053)	.669
Fever	1.088 (0.703–1.683)	.705	1.074 (0.497–2.319)	.856
Sore throat	0.279 (0.092–0.844)	**.024**	0.276 (0.082–0.921)	**.036**
Chest pain	1.984 (0.790–4.986)	.145	1.760 (0.649–4.772)	.267
Dyspnea	1.419 (0.979–2.055)	.064	1.840 (0.976–3.470)	.060
Fatigue	1.368 (0.874–2.143)	.170	1.384 (0.717–2.672)	.332
Nasal congestion	0.874 (0.216–3.536)	.850	0.636 (0.064–6.365)	.700
Headache	1.258 (0.393–4.023)	.699	0.742 (0.089–6.154)	.782
Myalgia/arthralgia	0.795 (0.391–1.616)	.526	0.445 (0.159–1.247)	.123
Nausea	3.130 (0.904–10.841)	.072	2.437 (0.494–12.013)	.274
Vomiting	1.324 (0.452–3.879)	.608	1.764 (0.372–8.374)	.475
Diarrhea	0.472 (0.172–1.293)	.144	0.474 (0.153–1.469)	.196
Abdominal pain	1.510 (0.500–4.563)	.465	1.300 (0.260–6.501)	.749
Concomitant acute liver dysfunction				
Present	2.512 (1.234–5.117)	**.011**	1.398 (0.639–3.056)	.402
Absent	1.000	–	1.000	–

Bold text indicates statistically significant data.

AKI = acute kidney injury, CI = confidence interval, CKD = chronic kidney disease, CLD = chronic liver disease, COPD = chronic obstructive pulmonary disease, HDU = high dependency unit, ICU = intensive care unit, IHD = ischemic heart disease, OR = odds ratio.

**Table 3:** Multivariable analysis of risk factors leading to mortality (n = 1069).

Factors	Univariate		Multivariate	
	Crudes OR (95% CI)	*P* value	Adjusted OR (95% CI)	*P* value
Age (in yr)				
<25	0.352 (0.127–0.977)	**.045**	0.552 (0.155–1.970)	.360
26–50	0.273 (0.154–0.485)	**<.001**	0.292 (0.146–0.583)	**<.001**
51–75	0.673 (0.400–1.134)	.137	0.546 (0.295–1.009)	.054
>75	1.000	–	1.000	–
Gender				
Male	1.029 (0.771–1.374)	.846	0.917 (0.631–1.332)	.650
Female	1.000	–	1.000	–
BMI (kg/m^2^)				
<18.5	1.200 (0.687–2.097)	.522	1.088 (0.602–1.722)	.771
18.5–24.9	1.180 (0.701–1.985)	.534	1.060 (0.643–1.720)	.831
25.0–29.9	1.191 (0.688–2.061)	.532	1.092 (0.699–1.962)	.757
≥30.0	1.000	–	1.000	–
Travel history				
Present	0.587 (0.131–2.627)	.486	0.599 (0.132–2.717)	.506
Absent	1.000	–	1.000	–
Profession				
Healthcare worker	0.898 (0.190–4.246)	.892	1.271 (0.258–6.253)	.768
Non-medical	1.000	–	1.000	–
Hospital stay				
ICU/HDU	7.395 (5.006–10.925)	**<.001**	2.976 (1.913–4.629)	**<.001**
Non-ICU/HDU	1.000	–	1.000	–
Mode of ventilation				
Invasive	10.583 (7.457–15.019)	**<.001**	9.210 (6.029–14.068)	**<.001**
Noninvasive	1.000	–	1.000	–
Comorbidities				
Diabetes	2.238 (1.128–4.440)	**.021**	1.971 (0.955–4.066)	.066
Hypertension	2.673 (1.309–5.462)	**.007**	2.057 (0.962–4.400)	.063
COPD	5.553 (0.759–40.627)	.091	5.264 (0.695–39.893)	.108
IHD	2.553 (1.031–6.321)	**.043**	1.190 (0.730–4.997)	.187
CKD	0.707 (0.201–2.487)	.589	0.250 (0.033–1.921)	.183
CLD	1.340 (0.146–12.309)	.796	2.313 (0.209–25.634)	.494
Asthma	1.199 (0.249–5.767)	.821	1.250 (0.255–6.125)	.783
Clinical features				
Dry cough	0.884 (0.567–1.376)	.584	1.680 (0.636–4.442)	.295
Productive cough	2.029 (1.032–3.991)	**.040**	3.232 (1.000–10.451)	**.050**
Fever	0.879 (0.528–1.462)	.619	1.020 (0.322–3.230)	.973
Sore throat	0.426 (0.096–1.882)	.260	0.271 (0.046–1.581)	.147
Chest pain	4.125 (1.565–10.875)	**.004**	3.531 (1.174–10.625)	**.025**
Dyspnea	2.047 (0.979–4.283)	.057	4.583 (1.547–13.584)	**.006**
Fatigue	1.447 (0.863–2.427)	.161	1.641 (0.713–3.779)	.244
Nasal congestion	2.057 (0.505–8.370)	.314	3.904 (0.138–10.260)	.424
Headache	0.362 (0.046–2.836)	.333	0.437 (0.014–13.629)	.637
Myalgia/arthralgia	0.593 (0.226–1.561)	.290	0.341 (0.063–1.843)	.211
Nausea	1.539 (0.401–5.908)	.530	1.315 (0.154–11.201)	.802
Vomiting	0.305 (0.309–2.356)	.255	0.935 (0.030–9.439)	.970
Lethargy	1.500 (0.146–15.461)	.733	2.206 (0.163–29.785)	.551
Abdominal pain	0.332 (0.043–2.581)	.292	0.426 (0.014–12.513)	.621

Bold text indicates statistically significant data.

AKI = acute kidney injury, BMI = body mass index, CI = confidence interval, CKD = chronic kidney disease, CLD = chronic liver disease, COPD = chronic obstructive pulmonary disease, HDU = high dependency unit, ICU = intensive care unit, IHD = ischemic heart disease, OR = odds ratio.

**Figure 2.**
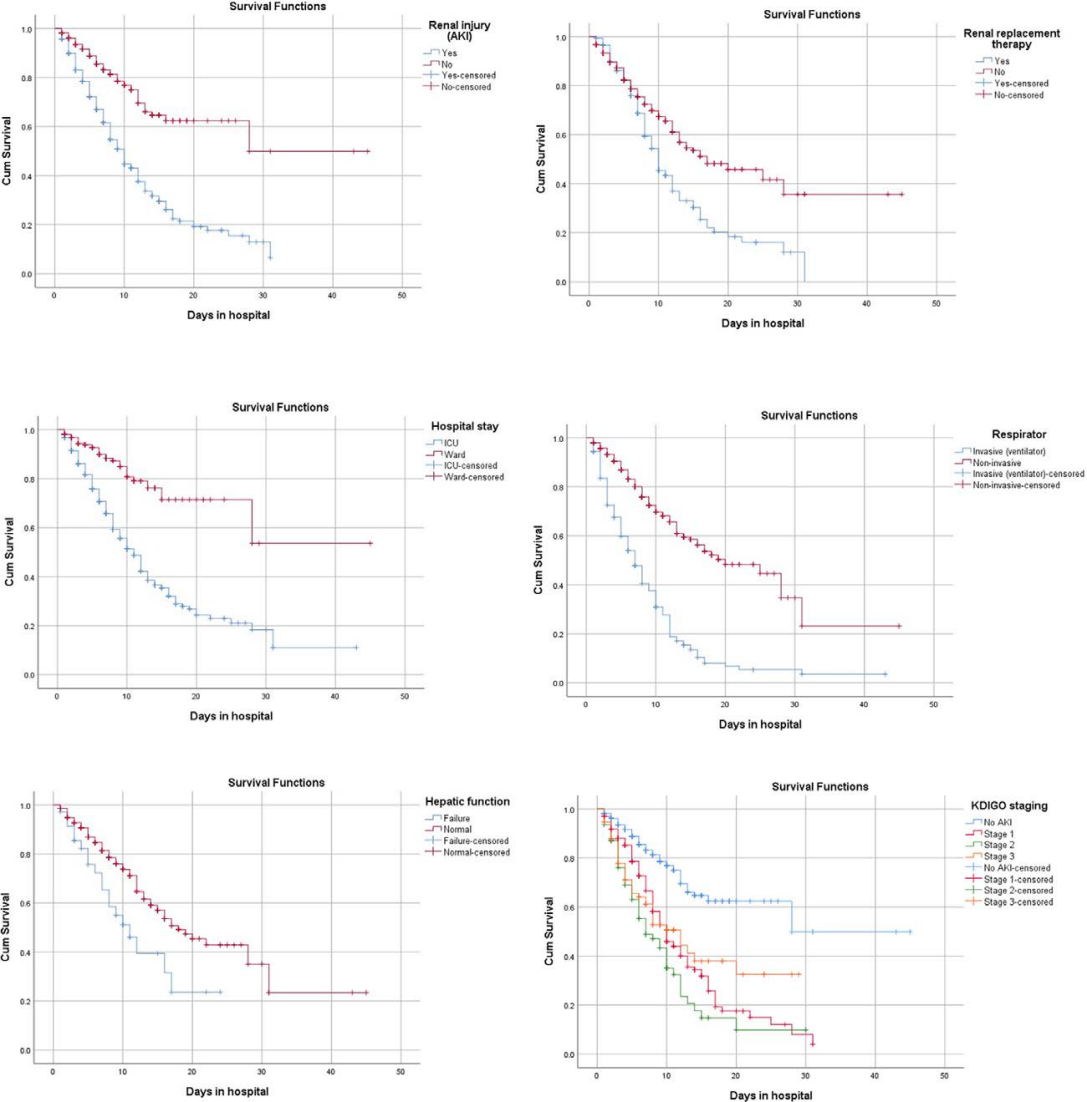
Kaplan–Meier curves of significant associated factors of AKI and mortality. AKI = acute kidney injury, ICU = intensive care unit.

Enlisted are the laboratory markers that were significantly rising from admission to discharge of AKI patients: neutrophils; NLR; creatinine; chloride; sodium; ferritin and procalcitonin; while platelets, lymphocytes, and monocytes tend to decline. In the mortality group (n = 290), TLC, neutrophils, NLR, urea, creatinine, chloride, sodium, potassium, ALT, PT/INR, LDH, ferritin, procalcitonin, and D-dimer were rising during hospital stay while lymphocytes, monocytes, calcium tends to decline (Table 4 and Table S1, Supplemental Digital Content, http://links.lww.com/MD/I446). Table S2, Supplemental Digital Content, http://links.lww.com/MD/I447 and Table S3, Supplemental Digital Content, http://links.lww.com/MD/I448 show a comparison of laboratory markers at admission and discharge respectively. Table S4, Supplemental Digital Content, http://links.lww.com/MD/I449 gives details of sensitivity analysis of screened data and missing cohort. Figure 2 showcased Kaplan–Meier survival curves of in-hospital mortality and AKI associated factors during the length of hospital stay. Significant factors of mortality were ICU care (*P* < .001), ventilator support (*P* < .001), renal replacement therapy (*P* < .001), concomitant liver dysfunction (*P* = .005), AKI (*P* < .001), and KDIGO staging (*P* < .001).

**Table 4:** Longitudinal analysis of biochemical markers from entry point (admission) to exit point (hospital discharge or death) during hospital stay among AKI group and disease outcomes (n = 1069).

Laboratory markers	Mean levels at admission	AKI		No AKI		Survived		Mortality	
		↑	↓	↑	↓	↑	↓	↑	↓
Hemoglobin (g/dL)	11.98 ± 2.35	36.3%	63.7%	40.1%	59.9%	39.2%	60.8%	35.2%	64.8%
MCV (fL)	84.56 ± 8.22	69.0%	31.0%	72.3%	27.7%	69.2%	30.8%	73.4%	26.6%
TLC (×10^9^/L)	11.51 ± 6.70	64.2%	35.8%	58.5%	41.5%	**58.3%**	**41.7%**	**67.6%**	**32.4%**
Platelets (×10^9^/L)	231.61 ± 109.08	**45.2%**	**54.8%**	**62.9%**	**37.1%**	**41.5%**	**58.5%**	**60.5%**	**39.5%**
Neutrophils (%)	76.59 ± 13.39	**63.8%**	**36.2%**	**46.1%**	**53.9%**	**46.2%**	**53.8%**	**72.7%**	**27.3%**
Lymphocytes (%)	17.02 ± 11.30	**45.9%**	**54.1%**	**56.9%**	**43.1%**	**56.7%**	**43.3%**	**40.4%**	**59.6%**
NLR	8.65 ± 10.34	**61.0%**	**39.0%**	**45.7%**	**54.3%**	**46.2%**	**53.8%**	**68.1%**	**31.9%**
Monocytes (%)	5.11 ± 3.56	**47.3%**	**52.7%**	**62.2%**	**37.8%**	**62.0%**	**38.0%**	**41.5%**	**58.5%**
Eosinophils (%)	1.17 ± 1.93	52.6%	47.4%	60.0%	40.0%	58.1%	41.9%	53.8%	46.2%
Basophils (%)	0.13 ± 0.41	**12.5%**	**87.5%**	**75.0%**	**25.0%**	25.0%	75.0%	37.5%	62.5%
Urea (mg/dL)	59.09 ± 55.55	70.5%	29.5%	63.6%	36.4%	**60.2%**	**39.8%**	**82.9%**	**17.1%**
Creatinine (mg/dL)	1.82 ± 2.58	**59.1%**	**40.9%**	**36.3%**	**63.7%**	**38.9%**	**61.1%**	**69.1%**	**30.9%**
Chloride (mg/dL)	103.24 ± 6.09	**66.9%**	**33.1%**	**57.4%**	**42.6%**	**56.1%**	**43.9%**	**75.4%**	**24.6%**
Sodium (mg/dL)	138.09 ± 6.08	**76.4%**	**23.6%**	**66.5%**	**33.5%**	**66.1%**	**33.9%**	**83.8%**	**16.2%**
Potassium (mg/dL)	4.14 ± 0.86	52.2%	47.8%	48.3%	51.7%	**44.2%**	**55.8%**	**63.1%**	**36.9%**
Bicarbonate (mg/dL)	20.45 ± 4.19	72.5%	27.5%	69.2%	30.8%	72.1%	27.9%	68.5%	31.5%
Magnesium (mg/dL)	2.19 ± 0.49	63.9%	36.1%	61.8%	38.2%	60.2%	39.8%	68.9%	31.3%
Phosphate (mg/dL)	4.09 ± 2.08	62.6%	37.4%	52.9%	47.1%	57.1%	42.9%	63.9%	36.1%
Calcium (mg/dL)	8.12 ± 0.81	46.8%	53.2%	62.5%	37.5%	**59.4%**	**40.6%**	**40.0%**	**60.0%**
Total bilirubin (mg/dL)	0.85 ± 1.31	56.9%	43.1%	75.0%	25.0%	57.5%	42.5%	68.1%	31.9%
Direct bilirubin (mg/dL)	0.50 ± 0.96	58.5%	41.5%	63.6%	36.4%	62.5%	37.5%	59.4%	40.6%
Indirect bilirubin (mg/dL)	0.36 ± 0.39	55.7%	44.3%	74.4%	25.6%	53.8%	46.2%	69.2%	30.8%
ALT (IU/L)	75.50 ± 188.24	61.5%	38.5%	47.8%	52.2%	**44.3%**	**55.7%**	**75.6%**	**24.4%**
AST (IU/L)	117.46 ± 457.54	60.9%	39.1%	45.5%	54.5%	50.0%	50.0%	62.5%	37.5%
ALP (IU/L)	120.72 ± 104.72	71.4%	28.6%	63.6%	36.4%	64.2%	35.8%	75.0%	25.0%
GGT (IU/L)	87.03 ± 91.13	50.8%	49.2%	61.9%	38.1%	60.0%	40.0%	47.5%	52.5%
PT (s)	12.66 ± 6.29	59.7%	40.3%	53.8%	46.2%	**38.9%**	**61.1%**	**74.3%**	**25.7%**
INR	1.34 ± 1.44	62.3%	37.7%	60.0%	40.0%	**44.8%**	**55.2%**	**76.5%**	**23.5%**
APTT (s)	34.85 ± 27.75	66.7%	33.3%	60.0%	40.0%	56.3%	43.8%	76.9%	23.1%
Fibrinogen (mg/dL)	469.56 ± 219.73	–	–	–	–	–	–	–	–
CRP (mg/L)	14.44 ± 11.74	34.7%	65.3%	31.3%	68.7%	31.6%	68.4%	35.3%	64.7%
Ferritin (ng/mL)	1584.73 ± 4493.76	**64.4%**	**35.6%**	**44.5%**	**55.5%**	**44.0%**	**56.0%**	**76.6%**	**23.4%**
LDH (U/L)	636.83 ± 689.04	56.0%	44.0%	49.8%	50.2%	**47.9%**	**52.1%**	**62.4%**	**37.6%**
Procalcitonin (ng/mL)	2.98 ± 9.50	**57.6%**	**42.4%**	**43.2%**	**56.8%**	**42.0%**	**58.0%**	**68.0%**	**32.0%**
D-Dimer (µg/mL)	6.00 ± 10.68	59.5%	40.5%	51.1%	48.9%	**50.3%**	**49.7%**	**66.2%**	**33.8%**
Troponin I (pg/mL)	423.24 ± 2435.17	52.2%	47.8%	41.7%	58.3%	39.1%	60.9%	57.1%	42.9%
Pro-BNP (pg/mL)	6157.07 ± 20106.99	72.7%	27.3%	50.0%	50.0%	55.6%	44.4%	75.0%	25.0%
ESR (mm/h)	50.05 ± 38.78	–	–	–	–	–	–	–	–
Albumin (g/dL)	2.82 ± 0.70	25.0%	75.0%	100.0%	0.0%	37.5%	62.5%	50.0%	50.0%

Bold text indicates statistically significant data.

AKI = acute kidney injury, ALP = alkaline phosphatase, ALT = alanine aminotransferase, APTT = activated partial thromboplastin time, AST = aspartate aminotransferase, BNP = B-type natriuretic peptide, CRP = C-reactive protein, ESR = erythrocyte sedimentation rate, GGT = gamma glutamyl transferase, INR = international normalized ratio, LDH = lactate dehydrogenase, MCV = mean corpuscular volume, NLR = neutrophil to lymphocyte ratio, PT = prothrombin time, TLC = total leukocyte count, ↑ increased from admission to discharge/death, ↓ decreased from admission to discharge/death.

## 4. Discussion

The event of AKI in patients with COVID-19 has been accounted for all around the world. Prior studies from China have demonstrated to be represented the event of AKI in COVID-19 patients is among 0.5% to 7%,^[13]^ and from all around the world about 28% to 45%.^[14]^ Furthermore, a retrospective study conducted in January 2020 in China on COVID-19 patients shows the presence of AKI in just 6 patients of around 0.5% out of 1099 patients.^[15]^ It was further revealed in a retrospective Cohort study by Yang et al that the mortality of 61.5% and occurrence of AKI in 29% among the 52 patients, was related to the complication of pneumonia.^[16]^ Moreover, in another review the AKI incident in COVID-19 patients went from 3.5% in moderately sick patients to 42.9% in severely ill patients further affirming the higher demise rate.^[17]^

AKI has been one of the significant reasons for higher mortality in patients with COVID-19.^[18]^ In relation to time, it was further reviewed in a study that 72.6% of patients were found to be having an incidence of AKI at the time of presentation. They likewise found an expanded risk of AKI determination at the hour of initiation of mechanical ventilation and subsequently suggested that the commonest cause of AKI has been the ischemic acute tubular injury. Furthermore, it was seen in the United States that the patients conceded with Coronavirus have a higher event of AKI in connection with other conceded patients for sepsis (22%), and for community-acquired pneumonia (34%).^[19]^ In another evaluation in 2021, the analysis showed no association of diabetes and hypertension with severe AKI, however, it was found to have an association with CKD.^[20]^ In our study, we found hypertension was more consistently associated with AKI as compared to Diabetes (refer to Table 2).

The mechanism of AKI in patients experiencing Coronavirus is still not clear, although, in a study by Su et al,^[21]^ intense acute tubular damage has been found in a series of postpartum patients experiencing COVID-19. Patients having extensive AKI close to stage 3 or more and requiring mechanical ventilation are viewed to be related with mortality.^[13]^ In another study by Wang et al, it was observed that the values of blood urea and creatinine were altogether higher in patients who couldn’t survive the illness as opposed to the survivors.^[22]^ Furthermore, another study conducted on patients with ventilators shows the presence of AKI in almost every other patient.^[23]^ Most patients experiencing AKI with COVID-19 were found to have a deranged renal profile at the time of discharge similar to our findings.

There can be gender and racial disparities of AKI in COVID-19 patients as described previously,^[24]^ however, we did not take racial differences into account as most of our study participants were from South Asian locality, but gender had no predisposition to AKI in our cohort. On the contrary, Soheili et al^[25]^ found females more prone to AKI in their cohort of 946 patients. In another cohort, the mortality rate of COVID-19 patients with AKI was exceeding 56%.^[26]^ Another cohort from our neighboring country India, shows 2650 hospitalized patients with only 7.2% developing AKI, and the majority were males.^[14]^ They also had a similar frequency of comorbidities comparable with our study like diabetes mellitus in 72.1%, hypertension in 66.8%, heart disease in 30%, and chronic kidney disease in 22.6%. While reviewing other local data, 41 out of 334 patients were reportedly undergoing hemodialysis for developing AKI and 30 of them died.^[27]^ In a pooled meta-analysis, there were higher odds of AKI in previous CKD patients, and who were on renal replacement therapy, and severity of the disease was also higher in these patients, which was affirmed by our findings.^[28]^

A well-reported Turkish cohort also reproduces our findings of in-hospital mortality not influenced by preexisting CKD, or any AKI over CKD during the COVID-19 disease course.^[5]^ With respect to age, there is a bimodal distribution of AKI among COVID-19 sufferers, with relatively younger and then the elder being more prone to it.^[29]^ Pediatric age involvement is frequently reported with AKI during COVID-19 illness.^[30,31]^ However, in our cohort, we only found it associated with increasing age. Now concerning the presentation, Pitre et al^[32]^ gave their aspect of 57.6% of patients presented with AKI to the hospital, while the rest 42.4% developed AKI during in-hospital stay. Furthermore, these in-hospital events can be attributed to the disease course, development of cytokine release storm,^[33]^ or drug-induced renal injury related to COVID-19 therapy.^[34]^ Despite this aspect could not be covered by our analysis, concomitant liver injury influencing AKI is another contributory event that can be attributed to multi-organ failure associated with COVID-19. The same findings were reproduced by a large single-center Iranian cohort.^[35]^

Accounting for the waxing and waning pattern of COVID-19 cases, spikes, and waves, many studies have reported discrepancies in AKI over time with COVID-19. The first of these studies showed the incidence of AKI being significantly lower in the second wave.^[36]^ But the Italian cohort showed similar rates of AKI in both waves of COVID-19.^[37]^ While, we reported previously in our comparison of waves that similar rises in serum urea levels were observed during both waves, while peak levels were attained early in the second wave, despite creatinine was deranged equally in both waves.^[38]^ Coming to laboratory derangements, Ghosn and his colleagues did not find an association between inflammatory (interleukin-6, C-reactive protein, and ferritin) or thrombotic (D-dimer and fibrinogen) markers with severe AKI after adjustment for potential confounders.^[39]^ In another analysis, there was a significant relationship between the KDIGO AKI classification stage and serum levels of ferritin, interleukin-6, and procalcitonin.^[40]^ This was also the case in our cohort as serum ferritin and procalcitonin were tend to be higher throughout the hospital course but we did not have the facility to record interleukin-6 levels at all of these studied centers. This was one potential confounder in our study, that is, lack of documentation of cytokine release storm associated with COVID-19. Other potential limitations include lack of determining urinary markers for renal injury like proteinuria as outlined by Lombardi et al in their study.^[41]^ We relied upon serum markers and KDIGO classification,^[11]^ for AKI determination but without documenting the categorization of patients into stages of AKI which is been frequently reported in the literature. However, we were not aiming the external validity for staging AKI because of the main objectives revolved around the determination of AKI with respect to symptomology, clinical profiles, and laboratory markers in our cohort. Since our study was more focused on the inclusion of patients with a prolonged hospital stay, we have discrepancies in our intensive care to non-intensive patient distribution within the groups (having more ICU inclusions). The information about COVID-19 infection in this study is limited, for instance, variants. Latent period and severity also differ by the type of virus variants. Also, percentage of population underwent vaccination might also affects the severity of disease.

In conclusion, the AKI in patients with COVID-19 resulted in the severe disease course and in-hospital mortality. Optional results were recovery from AKI and the role of laboratory markers throughout the hospital stay and at the time of discharge. Future studies should investigate the role of renal replacement therapy as primary management for AKI associated with COVID-19. Lastly, determining the rate of permanent dialysis patients would also be a future prospect to study in COVID-19 patients, since we could not followed up our patients for over 90 days due to retrospective study design and limitations.

## Author contributions

**Conceptualization:** Muhammad Nadeem Ahsan.

**Data curation:** Sadia Iqbal, Mohammed Akram, Basmah Fayyaz.

**Formal analysis:** Muhammad Sohaib Asghar, Sarosh Alvi.

**Project administration:** Zohaib Yousaf.

**Resources:** Irfan Ullah.

**Software:** Muhammad Sohaib Asghar.

**Supervision:** Haris Alvi.

**Writing – original draft:** Muhammad Sohaib Asghar, Syeda Ghazala Irshad.

## Supplementary Material








