# Supplementary material for: Outcomes of COVID-19 patients with acute kidney injury and longitudinal analysis of laboratory markers during the hospital stay: A multi-center retrospective cohort experience from Pakistan
Source: Medicine (Baltimore). 2023 Feb 10;102(6):e32919. doi: 10.1097/MD.0000000000032919 (PMC9907899; doi:10.1097/MD.0000000000032919)
Supplement: Supplementary file 1 [file medi-102-e32919-s001.pdf]

Supplementary Table 1. Chi-square significance (P-values) of longitudinal comparison of biochemical markers from entry point (admission) to exit point (hospital discharge or death) during hospital stay among study groups from Table 4 analysis.

**Laboratory markers**	**Mean levels at Discharge/death**	**AKI vs No AKI group**	**Survived vs Mortality group**
Hemoglobin (g/dL)	11.43±2.21	0.365	0.361
MCV (fL)	86.26±8.84	0.472	0.389
TLC ($x10^9$/L)	14.13±8.91	0.155	**0.031**
Platelets ($x10^9$/L)	248.08±135.76	**<0.001**	**<0.001**
Neutrophils (%)	78.78±13.91	**<0.001**	**<0.001**
Lymphocytes (%)	14.80±11.78	**0.007**	**<0.001**
NLR	13.02±16.32	**<0.001**	**<0.001**
Monocytes (%)	4.96±2.62	**0.002**	**<0.001**
Eosinophils (%)	1.00±1.77	0.543	0.727
Basophils (%)	0.14±0.66	**0.030**	0.665
Urea (mg/dL)	89.84±70.52	0.081	**<0.001**
Creatinine (mg/dL)	2.10±2.03	**<0.001**	**<0.001**
Chloride (mg/dL)	104.40±7.35	**0.027**	**<0.001**
Sodium (mg/dL)	141.63±7.55	**0.013**	**<0.001**
Potassium (mg/dL)	4.20±0.98	0.383	**<0.001**
Bicarbonate (mg/dL)	22.56±4.95	0.411	0.389
Magnesium (mg/dL)	2.32±0.71	0.795	0.257
Phosphate (mg/dL)	4.92±2.78	0.241	0.390
Calcium (mg/dL)	7.90±0.84	0.134	**0.046**
Total bilirubin (mg/dL)	0.95±1.09	0.053	0.265
Direct bilirubin (mg/dL	0.60±0.87	0.588	0.751
Indirect bilirubin (mg/dL)	0.36±0.31	0.051	0.115
ALT (IU/L)	98.82±181.19	0.152	**0.001**
AST (IU/L)	128.98±306.86	0.112	0.208
ALP (IU/L)	142.15±108.14	0.394	0.245
GGT (IU/L)	83.42±64.40	0.262	0.211
PT (sec)	13.85±4.93	0.627	**0.003**
INR	1.42±1.36	0.892	**0.010**
APTT (sec)	41.97±38.16	0.775	0.244
Fibrinogen (mg/dL)	404.20±317.81	-	-
CRP (mg/L)	10.84±10.46	0.414	0.421
Ferritin (ng/mL)	1854.67±4169.78	**<0.001**	**<0.001**
LDH (U/L)	686.92±853.94	0.228	**0.011**
Procalcitonin (ng/mL)	6.18±16.16	**0.040**	**<0.001**
D-Dimer (mcg/mL)	6.00±10.68	0.081	**0.002**
Troponin I (pg/mL)	955.08±2760.52	0.517	0.180
Pro-BNP (pg/mL)	8279.08±10388.65	0.349	0.402
ESR (mm/Hour)	63.60±40.34	-	-
Albumin (g/dL)	2.61±0.38	0.053	0.747

AKI: Acute kidney injury, MCV: Mean corpuscular volume, TLC: Total leukocyte count, NLR: Neutrophil to lymphocyte ratio, ALT: Alanine aminotransferase, AST: Aspartate aminotransferase, ALP: Alkaline phosphatase, GGT: Gamma glutamyl transferase, PT: Prothrombin time, INR: International normalized ratio, APTT: Activated partial thromboplastin time, CRP:

C-reactive protein, LDH: Lactate dehydrogenase, BNP: B-type natriuretic peptide, ESR: Erythrocyte sedimentation rate, ↑ Increased from admission to discharge/death ↓ Decreased from admission to discharge/death. **(Bold text shows significance).**
