# Supplementary material for: Outcomes of COVID-19 patients with acute kidney injury and longitudinal analysis of laboratory markers during the hospital stay: A multi-center retrospective cohort experience from Pakistan
Source: Medicine (Baltimore). 2023 Feb 10;102(6):e32919. doi: 10.1097/MD.0000000000032919 (PMC9907899; doi:10.1097/MD.0000000000032919)
Supplement: Supplementary file 2 [file medi-102-e32919-s002.pdf]

Supplementary Table 2. Comparative analysis of biochemical markers on admission among AKI vs non-AKI group and survived vs mortality group (n=1069) {Significance calculated by non-parametric distribution–Mann Whitney U-test}.

Laboratory markers	AKI (n=431)	No AKI (n=598)	P-value	Survived (n=779)	Mortality (n=290)	P-value
Hemoglobin (g/dL)	11.40±2.52	12.38±2.13	**<0.001**	12.06±2.33	11.75±2.40	0.053
MCV (fL)	85.54±8.36	83.70±7.96	**0.012**	84.26±8.11	85.35±8.49	0.182
TLC (x10^9/L)	13.34±7.28	10.25±5.97	**<0.001**	10.56±6.01	14.08±7.71	**<0.001**
Platelets (x10^9/L)	221.29±112.36	238.71±106.16	**0.002**	232.63±108.40	228.85±111.06	0.917
Neutrophils (%)	80.76±12.74	73.81±13.01	**<0.001**	74.70±13.08	81.71±12.87	**<0.001**
Lymphocytes (%)	12.92±10.16	19.79±11.15	**<0.001**	18.77±11.16	12.30±10.28	**<0.001**
NLR	11.55±12.05	6.67±8.45	**<0.001**	7.20±8.93	12.57±12.63	**<0.001**
Monocytes (%)	5.21±2.68	4.96±4.45	**0.006**	5.11±2.62	5.12±5.17	**0.014**
Eosinophils (%)	0.97±1.96	1.34±1.92	**0.001**	1.37±2.01	0.80±1.73	**0.001**
Basophils (%)	0.11±0.31	0.15±0.49	0.735	0.16±0.47	0.07±0.26	0.085
Urea (mg/dL)	97.00±66.76	31.50±16.81	**<0.001**	52.26±53.02	77.58±58.05	**<0.001**
Creatinine (mg/dL)	3.02±3.43	0.95±1.11	**<0.001**	1.70±2.64	2.13±2.41	**<0.001**
Chloride (mg/dL)	103.43±7.41	103.10±4.95	0.748	103.41±5.78	102.80±6.84	0.051
Sodium (mg/dL)	137.97±7.53	138.17±4.80	**0.038**	138.14±5.64	137.96±7.13	0.170
Potassium (mg/dL)	4.45±1.08	3.91±0.56	**<0.001**	4.11±0.83	4.21±0.94	0.058
Bicarbonate (mg/dL)	18.85±4.80	21.60±3.23	**<0.001**	20.83±3.83	19.44±4.85	**<0.001**
Magnesium (mg/dL)	2.26±0.57	2.08±0.33	**0.012**	2.18±0.51	2.22±0.46	0.194
Phosphate (mg/dL)	4.58±2.37	3.30±1.12	**<0.001**	3.98±2.18	4.34±1.86	**0.023**
Calcium (mg/dL)	8.01±0.84	8.30±0.72	**0.035**	8.20±0.82	7.99±0.78	**0.035**
Total bilirubin (mg/dL)	0.99±1.56	0.71±0.98	0.371	0.70±0.93	1.20±1.90	**0.001**
Direct bilirubin (mg/dL	0.63±1.18	0.37±0.66	**0.043**	0.40±0.67	0.73±1.42	**0.003**
Indirect bilirubin (mg/dL)	0.38±0.44	0.33±0.32	0.847	0.31±0.32	0.45±0.49	**0.005**
ALT (IU/L)	95.34±247.29	55.77±96.44	0.056	66.73±112.43	78.92±210.68	0.130
AST (IU/L)	168.33±630.74	65.23±108.60	**0.050**	105.51±252.81	122.67±522.83	**<0.001**
ALP (IU/L)	130.73±100.91	110.58±107.84	**0.005**	117.47±89.73	128.24±133.36	0.631
GGT (IU/L)	92.73±96.40	81.15±85.26	0.156	83.64±83.82	94.77±106.02	0.253
PT (sec)	13.54±7.85	11.38±2.24	**0.003**	12.49±5.28	13.03±8.14	0.379
INR	1.38±1.21	1.29±1.76	**0.036**	1.20±0.60	1.65±2.39	0.264
APTT (sec)	37.95±32.16	30.09±18.38	0.051	34.56±28.12	35.55±27.18	0.273
Fibrinogen (mg/dL)	470.47±211.68	468.00±243.63	0.914	448.46±183.90	485.70±248.01	0.660
CRP (mg/L)	17.04±11.37	12.57±11.69	**<0.001**	13.00±11.48	18.11±11.63	**<0.001**
Ferritin (ng/mL)	2138.30±5411.42	1142.92±3555.36	**<0.001**	1505.90±4651.24	1766.85±4113.08	**0.012**
LDH (U/L)	774.94±943.91	524.33±314.43	**<0.001**	545.25±445.86	846.92±1024.06	**<0.001**
Procalcitonin (ng/mL)	4.70±11.75	1.61±6.96	**<0.001**	2.19±8.12	4.74±11.87	**<0.001**
D-Dimer (mcg/mL)	7.96±12.22	4.19±8.72	**<0.001**	4.29±7.94	9.69±14.34	**<0.001**
Troponin I (pg/mL)	652.89±3148.27	95.59±293.69	**<0.001**	319.15±2169.54	575.29±2784.08	**<0.001**
Pro-BNP (pg/mL)	8601.99±25284.74	2112.33±4085.33	**<0.001**	5583.57±8671.21	6757.04±27466.73	**0.007**
ESR (mm/Hour)	62.28±45.60	36.21±23.87	0.118	42.53±28.34	61.52±49.36	0.397
Albumin (g/dL)	2.79±0.69	2.86±0.72	0.640	2.82±0.74	2.83±0.65	0.923

AKI: Acute kidney injury, MCV: Mean corpuscular volume, TLC: Total leukocyte count, NLR: Neutrophil to lymphocyte ratio, ALT: Alanine aminotransferase, AST: Aspartate aminotransferase, ALP: Alkaline phosphatase, GGT: Gamma glutamyl transferase, PT: Prothrombin time, INR: International normalized ratio, APTT: Activated partial thromboplastin time, CRP: C-reactive protein, LDH:

Lactate dehydrogenase, BNP: B-type natriuretic peptide, ESR: Erythrocyte sedimentation rate, ↑ Increased from admission to discharge/death ↓ Decreased from admission to discharge/death. **(Bold text indicate statistically significant data).**
