# Supplementary material for: Outcomes of COVID-19 patients with acute kidney injury and longitudinal analysis of laboratory markers during the hospital stay: A multi-center retrospective cohort experience from Pakistan
Source: Medicine (Baltimore). 2023 Feb 10;102(6):e32919. doi: 10.1097/MD.0000000000032919 (PMC9907899; doi:10.1097/MD.0000000000032919)
Supplement: Supplementary file 3 [file medi-102-e32919-s003.pdf]

Supplementary Table 3. Comparative analysis of biochemical markers on discharge/death among AKI vs non-AKI group and survived vs mortality group (n=1069) {Significance calculated by non-parametric distribution–Mann Whitney U-test}.

**Laboratory markers**	**AKI (n=431)**	**No AKI (n=598)**	P-value	**Survived (n=779)**	**Mortality (n=290)**	P-value
Hemoglobin (g/dL)	10.80±2.24	12.03±2.00	**<0.001**	11.52±2.20	11.24±2.22	0.062
MCV (fL)	87.45±8.67	85.04±8.69	**0.023**	85.54±8.56	87.79±9.26	**0.049**
TLC ($x10^9$/L)	16.85±10.03	11.57±6.80	**<0.001**	12.29±7.44	18.19±10.45	**<0.001**
Platelets ($x10^9$/L)	212.25±124.76	282.11±137.75	**<0.001**	265.44±137.06	210.42±125.20	**<0.001**
Neutrophils (%)	82.85±13.34	74.98±13.29	**<0.001**	75.84±13.64	85.19±12.25	**<0.001**
Lymphocytes (%)	11.33±11.27	18.04±11.24	**<0.001**	17.18±11.83	9.61±9.87	**<0.001**
NLR	8.45±10.80	17.85±19.48	**<0.001**	9.99±13.91	19.64±19.05	**<0.001**
Monocytes (%)	4.49±2.37	5.42±2.77	**<0.001**	5.45±2.71	4.00±2.14	**<0.001**
Eosinophils (%)	0.73±1.73	1.43±1.77	**0.003**	1.43±2.14	0.41±0.77	**<0.001**
Basophils (%)	0.05±0.23	0.31±1.06	**0.015**	0.22±0.87	0.06±0.24	0.157
Urea (mg/dL)	134.01±69.74	41.09±23.81	**<0.001**	68.38±56.74	138.04±74.68	**<0.001**
Creatinine (mg/dL)	3.25±2.25	0.83±0.26	**<0.001**	1.66±1.79	3.08±2.21	**<0.001**
Chloride (mg/dL)	106.12±8.40	102.43±5.27	**<0.001**	102.86±6.23	107.57±8.40	**<0.001**
Sodium (mg/dL)	143.54±8.57	139.42±5.39	**<0.001**	139.64±5.84	145.77±8.92	**<0.001**
Potassium (mg/dL)	4.48±1.12	3.87±0.67	**<0.001**	4.00±0.78	4.60±1.21	**<0.001**
Bicarbonate (mg/dL)	21.53±5.10	23.74±4.49	**<0.001**	23.18±4.57	21.27±5.44	**<0.001**
Magnesium (mg/dL)	2.36±0.57	2.24±0.94	**0.004**	2.24±0.76	2.46±0.58	**<0.001**
Phosphate (mg/dL)	5.59±2.85	3.51±2.02	**<0.001**	4.42±2.49	5.78±3.05	**0.002**
Calcium (mg/dL)	7.77±0.88	8.21±0.63	**0.004**	8.12±0.78	7.62±0.83	**<0.001**
Total bilirubin (mg/dL)	1.11±1.34	0.71±0.50	0.371	0.97±1.25	0.91±0.76	0.220
Direct bilirubin (mg/dL	0.75±1.06	0.39±0.40	**0.029**	0.61±1.00	0.58±0.59	0.064
Indirect bilirubin (mg/dL)	0.37±0.38	0.34±0.18	0.369	0.37±0.36	0.33±0.20	0.860
ALT (IU/L)	125.86±228.66	61.64±64.68	0.079	79.08±108.56	132.66±261.19	0.194
AST (IU/L)	175.07±391.66	61.84±40.11	0.126	118.58±330.86	147.24±262.33	**0.023**
ALP (IU/L)	153.27±128.31	126.19±68.00	0.100	141.70±127.63	142.92±62.79	0.256
GGT (IU/L)	83.84±57.97	82.79±73.71	0.449	81.36±58.63	84.65±67.99	0.841
PT (sec)	13.00±3.09	14.04±5.25	0.657	13.68±6.05	14.03±3.58	**0.021**
INR	1.19±0.31	1.47±1.49	0.247	1.31±0.33	1.53±1.92	**0.038**
APTT (sec)	41.35±37.27	44.06±42.55	0.562	39.82±41.68	43.90±35.30	0.092
Fibrinogen (mg/dL)	619.00±373.35	261.00±235.40	0.248	356.50±236.88	436.00±412.50	0.999
CRP (mg/L)	13.51±11.01	8.43±9.27	**<0.001**	8.87±9.87	15.35±10.40	**<0.001**
Ferritin (ng/mL)	2810.18±5784.82	1077.78±1724.14	**<0.001**	1120.19±1662.11	3733.90±7086.14	**<0.001**
LDH (U/L)	825.31±954.80	581.00±754.02	**<0.001**	533.42±376.73	1075.59±1420.81	**<0.001**
Procalcitonin (ng/mL)	8.93±19.26	2.67±10.04	**<0.001**	4.52±12.87	9.17±20.58	**<0.001**
D-Dimer (mcg/mL)	10.75±14.46	4.01±5.86	**<0.001**	4.61±6.66	13.08±16.39	**<0.001**
Troponin I (pg/mL)	1206.94±3113.74	398.90±1670.85	**0.025**	544.48±1929.80	1297.25±3282.32	**0.038**
Pro-BNP (pg/mL)	9502.00±11440.42	6444.71±8652.42	0.439	6079.78±7034.49	10890.75±13108.95	0.436
ESR (mm/Hour)	66.30±43.04	60.63±44.30	0.990	23.00±25.45	90.66±13.57	0.083
Albumin (g/dL)	2.53±0.34	3.10±0.14	0.054	2.69±0.37	2.41±0.38	0.178

AKI: Acute kidney injury, MCV: Mean corpuscular volume, TLC: Total leukocyte count, NLR: Neutrophil to lymphocyte ratio, ALT: Alanine aminotransferase, AST: Aspartate aminotransferase, ALP: Alkaline phosphatase, GGT: Gamma glutamyl transferase, PT: Prothrombin time, INR: International normalized ratio, APTT: Activated partial thromboplastin time, CRP: C-reactive protein, LDH:

Lactate dehydrogenase, BNP: B-type natriuretic peptide, ESR: Erythrocyte sedimentation rate, ↑ Increased from admission to discharge/death ↓ Decreased from admission to discharge/death.**(Bold text indicate statistically significant data).**
