# Supplementary material for: Outcomes of COVID-19 patients with acute kidney injury and longitudinal analysis of laboratory markers during the hospital stay: A multi-center retrospective cohort experience from Pakistan
Source: Medicine (Baltimore). 2023 Feb 10;102(6):e32919. doi: 10.1097/MD.0000000000032919 (PMC9907899; doi:10.1097/MD.0000000000032919)
Supplement: Supplementary file 4 [file medi-102-e32919-s004.pdf]

Supplementary Table 4. Variable wise missing data of the study population from the hospital's medical record (n=1069).

**Characteristics**	**Variables**	**Frequency (n)**	
		**Missing data**	**Available data**
Age	<25 years	n=30	n=1039
	26-50 years		
	51-75 years		
	>75 years		
Gender	Male	n=0	n=1069
	Female		
BMI	<18.5 kg/m^2	n=0	n=1069
	18.5-24.9 kg/m^2		
	25.0-29.9 kg/m^2		
	≥30.0 kg/m^2		
Travel history	Present	n=517	n=552
	Absent		
Known exposure	Family member	n=209	n=860
	Work place		
	Healthcare		
	Travelling		
	Unknown		
Profession	Healthcare worker	n=203	n=866
	Non-Medical		
Hospital stay	ICU/HDU	n=0	n=1069
	Non-ICU/HDU		
Mode of ventilation	Invasive	n=0	n=1069
	Non-invasive		
Comorbidities	Diabetes	n=255	n=814
	Hypertension	n=255	n=814
	COPD	n=253	n=816
	IHD	n=253	n=816
	CKD	n=256	n=813
	CLD	n=255	n=814
	Asthma	n=254	n=815
	Stroke	n=56	n=1013
	Hypothyroidism	n=56	n=1013
Clinical features	Dry cough	n=564	n=505
	Productive cough	n=565	n=504
	Fever	n=506	n=563
	Sore throat	n=249	n=820
	Chest pain	n=250	n=819
	Dyspnea	n=506	n=563
	Fatigue	n=506	n=563
	Nasal congestion	n=505	n=564
	Headache	n=505	n=564
	Myalgia/Arthralgia	n=505	n=564
	Nausea	n=506	n=563

	Vomiting	n=505	n=564
	Diarrhea	n=505	n=564
	Abdominal pain	n=506	n=563
	Lethargy	n=54	n=1015
Clinical outcomes	Mortality	n=0	n=1069
	Survived/Discharged		
Renal outcomes	AKI	n=40	n=1029
	No AKI		
Nature of renal injury	Resolving AKI	n=40	n=1069
	Worsening AKI		
Concomitant hepatic failure	Present	n=473	n=596
	Absent		
Renal replacement therapy	Hemodialysis	n=110	n=959
	No hemodialysis		
Hematological indices (at entry point/On admission)	Hemoglobin (g/dL)	n=100	n=969
	MCV (fL)	n=523	n=546
	TLC (x10^9/L)	n=35	n=1034
	Platelets (x10^9/L)	n=46	n=1023
	Neutrophils (%)	n=34	n=1035
	Lymphocytes (%)	n=36	n=1033
	NLR	n=36	n=1033
	Monocytes (%)	n=391	n=678
	Eosinophils (%)	n=788	n=281
	Basophils (%)	n=813	n=256
Hematological indices (at exit point/On discharge or death)	Hemoglobin (g/dL)	n=525	n=544
	MCV (fL)	n=669	n=400
	TLC (x10^9/L)	n=466	n=603
	Platelets (x10^9/L)	n=470	n=599
	Neutrophils (%)	n=468	n=601
	Lymphocytes (%)	n=470	n=599
	NLR	n=470	n=599
	Monocytes (%)	n=553	n=516
	Eosinophils (%)	n=921	n=148
	Basophils (%)	n=935	n=134
Renal markers (at entry point/On admission)	Urea (mg/dL)	n=46	n=1023
	Creatinine (mg/dL)	n=42	n=1027
	Chloride (mg/dL)	n=119	n=950
	Sodium (mg/dL)	n=116	n=953
	Potassium (mg/dL)	n=115	n=954
	Bicarbonate (mg/dL)	n=118	n=951
	Magnesium (mg/dL)	n=818	n=251
	Phosphate (mg/dL)	n=827	n=242
	Calcium (mg/dL)	n=900	n=169
Renal markers (at exit point/On discharge or death)	Urea (mg/dL)	n=501	n=568
	Creatinine (mg/dL)	n=498	n=571
	Chloride (mg/dL)	n=553	n=516
	Sodium (mg/dL)	n=549	n=520
	Potassium (mg/dL)	n=550	n=519
	Bicarbonate (mg/dL)	n=549	n=520

	Magnesium (mg/dL)	n=887	n=182
	Phosphate (mg/dL)	n=894	n=175
	Calcium (mg/dL)	n=939	n=130
Hepatic function enzymes (at entry point/On admission)	Total bilirubin (mg/dL)	n=756	n=313
	Direct bilirubin (mg/dL	n=756	n=313
	Indirect bilirubin (mg/dL)	n=775	n=294
	ALT (IU/L)	n=738	n=331
	AST (IU/L)	n=763	n=306
	ALP (IU/L)	n=761	n=308
	GGT (IU/L)	n=764	n=305
Hepatic function enzymes (at exit point/On discharge or death)	Total bilirubin (mg/dL)	n=956	n=113
	Direct bilirubin (mg/dL	n=956	n=113
	Indirect bilirubin (mg/dL)	n=960	n=109
	ALT (IU/L)	n=955	n=114
	AST (IU/L)	n=956	n=113
	ALP (IU/L)	n=957	n=112
	GGT (IU/L)	n=959	n=110
Coagulation profile (at entry point/On admission)	PT (sec)	n=841	n=228
	INR	n=849	n=220
	APTT (sec)	n=927	n=142
	Fibrinogen (mg/dL)	n=1039	n=30
Coagulation profile (at exit point/On discharge or death)	PT (sec)	n=986	n=83
	INR	n=987	n=82
	APTT (sec)	n=1012	n=57
	Fibrinogen (mg/dL)	n=1064	n=5
Inflammatory biomarkers (at entry point/On admission)	CRP (mg/L)	n=136	n=933
	Ferritin (ng/mL)	n=450	n=619
	LDH (U/L)	n=453	n=616
	Procalcitonin (ng/mL)	n=348	n=721
	D-Dimer (mcg/mL)	n=486	n=583
Inflammatory biomarkers (at exit point/On discharge or death)	CRP (mg/L)	n=551	n=518
	Ferritin (ng/mL)	n=619	n=395
	LDH (U/L)	n=616	n=385
	Procalcitonin (ng/mL)	n=842	n=227
	D-Dimer (mcg/mL)	n=633	n=436
Miscellaneous (at entry point/On admission)	Troponin I (pg/mL)	n=850	n=219
	Pro-BNP (pg/mL)	n=936	n=133
	ESR (mm/Hour)	n=1016	n=53
	Albumin (g/dL)	n=997	n=72
Miscellaneous (at exit point/On discharge or death)	Troponin I (pg/mL)	n=992	n=77
	Pro-BNP (pg/mL)	n=1034	n=35
	ESR (mm/Hour)	n=1064	n=5
	Albumin (g/dL)	n=1055	n=14

BMI: Body mass index, ICU: Intensive care unit, HDU: High dependency unit, COPD: Chronic obstructive pulmonary disease, CLD: Chronic liver disease, IHD: Ischemic heart disease, AKI: Acute kidney injury, CKD: chronic kidney disease, MCV: Mean corpuscular volume, TLC: Total leukocyte count, NLR: Neutrophil to lymphocyte ratio, ALT: Alanine aminotransferase, AST: Aspartate aminotransferase, ALP: Alkaline phosphatase, GGT: Gamma glutamyl transferase, PT: Prothrombin time, INR: International

normalized ratio, APTT: Activated partial thromboplastin time, CRP: C-reactive protein, LDH: Lactate dehydrogenase, BNP: B-type natriuretic peptide, ESR: Erythrocyte sedimentation rate, n: number of subjects.
